# Stress-Induced Locomotor Sensitization to Amphetamine in Adult, but not in Adolescent Rats, Is Associated with Increased Expression of ΔFosB in the Nucleus Accumbens

**DOI:** 10.3389/fnbeh.2016.00173

**Published:** 2016-09-12

**Authors:** Paulo E. Carneiro de Oliveira, Rodrigo M. Leão, Paula C. Bianchi, Marcelo T. Marin, Cleopatra da Silva Planeta, Fábio C. Cruz

**Affiliations:** ^1^Laboratory of Pharmacology, School of Pharmaceutical Sciences, São Paulo State University-UNESPAraraquara, Brazil; ^2^Joint Graduate Program in Physiological Sciences, UFSCar/UNESP, Faculdade de Odontologia de AraraquaraAraraquara, Brazil

**Keywords:** amphetamine, behavioral sensitization, stress, ΔFosB, adolescence

## Abstract

While clinical and pre-clinical evidence suggests that adolescence is a risk period for the development of addiction, the underlying neural mechanisms are largely unknown. Stress during adolescence has a huge influence on drug addiction. However, little is known about the mechanisms related to the interaction among stress, adolescence and addiction. Studies point to ΔFosB as a possible target for this phenomenon. In the present study, adolescent and adult rats (postnatal day 28 and 60, respectively) were restrained for 2 h once a day for 7 days. Three days after their last exposure to stress, the animals were challenged with saline or amphetamine (1.0 mg/kg i.p.) and amphetamine-induced locomotion was recorded. Immediately after the behavioral tests, rats were decapitated and the nucleus accumbens was dissected to measure ΔFosB protein levels. We found that repeated restraint stress increased amphetamine-induced locomotion in both the adult and adolescent rats. Furthermore, in adult rats, stress-induced locomotor sensitization was associated with increased expression of ΔFosB in the nucleus accumbens. Our data suggest that ΔFosB may be involved in some of the neuronal plasticity changes associated with stress induced-cross sensitization with amphetamine in adult rats.

## Introduction

Drug abuse often begins during adolescence, which is a period of ontogeny in which individuals exhibit some risk-taking behavior that may lead to unsafe decision associated with negative outcomes, such as substance use (Cavazos-Rehg et al., 2011). In rats, adolescence has been defined as period from postnatal day (P) 28 to P42 (Spear and Brake, 1983). At this period rats display adolescent-typical neurobehavioral characteristics (Teicher et al., 1995; Laviola et al., 1999; Spear, 2000b).

Several clinical studies indicate that adolescence is a more vulnerable period for the development of drug addiction (Spear, 2000a,b; Izenwasser and French, 2002). This greater vulnerability to addiction might be explained by different outcomes of drug administration between adolescents and adults (Collins and Izenwasser, 2002). For instance, the locomotor-stimulating properties of amphetamine and cocaine are lower in adolescents compared with adults (Laviola et al., 1999; Tirelli et al., 2003). Furthermore, adolescents relative to adult show greater intake of cocaine, acquire cocaine self-administration more rapidly and self-administer higher doses of amphetamine (Shahbazi et al., 2008; Wong et al., 2013). Although evidence shows that adolescence is a risk period for the development of addiction, the neural mechanisms are not well known.

Studies have demonstrated that adolescence is a sensitive period which may exacerbate a predisposition for the development of stress-induced physical and behavioral disorders (Bremne and Vermetten, 2001; Heim and Nemeroff, 2001; Cymerblit-Sabba et al., 2015). Studies in animal models evidenced that adolescents are particularly vulnerable to the negative consequences of stress. For example, adolescent rodents are more sensitive to stress-induced weight loss, reductions in food intake and anxiety-like behaviors than their adult counterparts (Stone and Quartermain, 1997; Doremus-Fitzwater et al., 2009; Cruz et al., 2012). Cymerblit-Sabba et al. (2015) showed that adolescent rats at P28–54, demonstrated more vulnerability to stress than when rats were subject to stress in other periods of life.

It is well established that stressful life events during the adolescence are an important factor for developing drug addiction (Laviola et al., 1999; Tirelli et al., 2003; Cruz et al., 2010). In rats, repeated episodes of stress can increase motor activity in response to an acute drug (Covington and Miczek, 2001; Marin and Planeta, 2004; Cruz et al., 2011); this phenomenon is termed behavioral cross-sensitization (Covington and Miczek, 2001; Miczek et al., 2008; Yap and Miczek, 2008) and is thought to reflect neuronal adaptation in the mesocorticolimbic system related to development of drug addiction (Robinson et al., 1985; Robinson and Berridge, 2008; Vanderschuren and Pierce, 2010). In adult rats, it is well established that stressful experiences in adulthood cause behavioral sensitization to drug of abuse (Miczek et al., 2008; Yap et al., 2015) and that the enhanced locomotor stimulant effect of cocaine can persist for several weeks as a result of neuroadaptations in the mesocorticolimbic dopamine pathway (Vanderschuren and Kalivas, 2000; Hope et al., 2006).

Acute or repeated stress-induced cross-sensitization has been associated with plasticity in mesocorticolimbic system (Miczek et al., 2008; Yap and Miczek, 2008; Yap et al., 2015). Molecular and cellular plasticity in the brain requires changes in gene expression (Nestler et al., 1999). Gene expression is controlled by a series of DNA-binding proteins known as transcription factors (Chen et al., 1995, 1997, 2000). Several transcription factors have been implicated in this regulation, such as ΔFosB, a splice variant of the *fosb* gene, which is usually stable protein that accumulates with chronic exposure to drug and stress (McClung et al., 2004). ΔFosB appears to be a particularly important agent for long-term modifications in the nervous system involved with addictive behaviors (Damez-Werno et al., 2012; Pitchers et al., 2013). Indeed, it has been demonstrated that Δ-FosB mediates long-lasting adaptations of the brain underlying addiction behaviors (McClung et al., 2004). It was found that Δ-FosB may be responsible for the increases in spine density and dendritic arborization subsequent to chronic cocaine administration (Kolb et al., 2003; Lee et al., 2006), Moreover, Δ-FosB appears to be one of the mechanisms responsible for the sensitized reactions to psychostimulant (McClung and Nestler, 2003).

Adolescent rodents show peculiarities in mesolimbic function and in their profiles of sensitization to psychostimulant drugs (Laviola et al., 2003; Tirelli et al., 2003). For instance, overexpression of dopamine receptor and greater dopamine storage in synapses are reported in the mesolimbic system of adolescent rats (Tirelli et al., 2003). Ontogenetic changes in the mesolimbic system underlying sensitization may lead to different levels of vulnerability to drug addiction. Although the molecular mechanisms associated to cross-sensitization between stress and drug have been characterized in adult animals, the consequences of stress exposure during adolescence on challenging effects of drug is less known.

For this propose, we assessed the level of ΔFosB, on accumbens of adult and adolescent rats following the locomotor cross-sensitization between repeated restraint stress and amphetamine.

## Materials and Methods

### Subjects

Male Wistar rats obtained from the animal breeding facility of the São Paulo State University—UNESP at postnatal day (P) 21. Groups of 3–4 animals were housed in plastic cages 32 (width) × 40 (length) × 16 (height) cm in a room maintained at 23 ± 2°C. Rats were kept in a 12:12 h light/dark cycle (lights on at 07:00 a.m.) and were allowed free access to food and water. Each animal was used only in one experimental procedure. All experiments were performed during the light phase between 8:00 a.m. and 5:00 p.m. Each experimental group consisted of 9–10 rats.

The experimental protocol was approved by the Ethical Committee for use of Human or Animal Subjects of the School of Pharmaceutical Science—UNESP (CEP-12/2008) and the experiments were conducted according to ethics principles of the Brazilian College of Animals’ Experimentation—(COBEA), based on NIH Guidelines for the Care and Use of Laboratory Animals.

### Drug

*d,l*-amphetamine (Sigma, St. Louis, MO, USA) dissolved in saline (0.9% NaCl).

### Repeated Stress Procedure

The animals were allocated into two groups: (1) no-stress; or (2) repeated restraint stress. Animals in the repeated restraint stress group were restrained in plastic cylinders [20.0 cm (length) × 5.5 cm (internal diameter) for adult rats; 17.0 cm (length) × 4.5 cm (internal diameter) for adolescent rats] 2 h daily for 7 days starting at 10:00 a.m.

Exposure to stress began on the P28 for adolescent or P60 adult rats. The control (non-stress) group consisted of animals of the same age left undisturbed except for cleaning the cages.

### Stress-Induced Cross-Sensitization to Amphetamine

Behavioral testing was conducted in commercially available (Columbus Instruments, Columbus, OH, USA) activity monitoring chambers, consisting of Plexiglas cages 44 (width) × 44 (length) × 16 (height). The chambers included 10 pairs of photocells beams, which were used to measure the horizontal locomotor activity. The consecutive interruption of two beams was recorded as one locomotion unit.

Three days after the last exposure to stress, adolescent or adult rats were transported from the animal facility to an experimental room where they were individually placed in an activity-monitoring chamber and left for 20 min for habituation. Following this period, rats from the control or stress groups received i.p. challenge injections of amphetamine (1.0 mg/kg) or saline (NaCl 0.9%) and were returned to the activity monitoring-chamber for another 40 min (*N* = 9–10 animals per group). Locomotor activity was recorded during these 40 min following the injections.

Adolescent and adult rats were tested, respectively, on P37 and P69.

### Collection of Brains

Immediately after the behavioral analysis, animals were transferred to an adjacent room, decapitated and their brains were rapidly removed (about 60–90 s) and frozen in isopentane on dry ice. Following this procedure, the brains were stored at −80°C until dissection of accumbens.

### Western Blotting Analysis of ΔFosB Expression

Frozen brains were serially sliced at 50 μm in the coronal plane until the interested brain areas in a cryostat (Leica CM 1850, Nussloch, Germany) kept at −20°C. Tissue punches (blunt 14-gauge needle for adults and 16-gauge for adolescents) were obtained from nucleus accumbens (Figure 2A) using the coordinates: approximately from +2, 1 mm to +1, 1 mm for accumbens relative to Bregma (Paxinos and Watson, 2006). Tissues were sonicated in 1% sodium dodecyl sulfate (SDS). Protein concentrations of the samples were determined using the method of Lowry (Bio-Rad Laboratories, Hercules, CA, USA). Sample protein concentrations were equalized by diluting with 1% SDS. Samples of 30 μg of protein were then subjected to SDS-polyacrylamide gel electrophoresis for 3 h at 200 V. Proteins were transferred electrophoretically to polyvinylidene fluoride (PVDF) membrane for immunoblotting Hybond LFP transfer membrane (GE Healthcare, Little Chanford, BU, UK) at 0.3 A for 3.5 h. Then PVDF membranes were blocked with 5% nonfat dry milk and 0.1% Tween 20 in Tris buffer (T-TBS, pH 7.5) for 1 h at room temperature and then incubated overnight at 4°C in fresh blocking buffer (2% nonfat dry milk and 0.1% Tween 20 in Tris buffer [T-TBS, pH 7.5]) containing the primary antibodies. ΔFosB levels were assessed using antibodies against FosB (1:1000; Cat # sc-48 Santa Cruz Biotechnology, Santa Cruz, CA, USA). After incubation with primary antibodies, blots were washed and incubated for 1 h with anti-rabbit secondary antibodies labeled with Cy5 fluorophore (anti-rabbit/1:3000; GE Healthcare, Little Chanford, BU, UK). Fluorescence was assessed using a fluorescence scanner TyphoonTrio^®^ (GE Healthcare, Little Chanford, BU, UK), and bands were quantified using suitable software (Image Quant^TM^ TL). The average of non-stressed saline group was considered 100% and data from the other groups were expressed as percentage of this control group.

The antibody used to detect FosB also binds with ΔFosB. However, we collected the brains 40 min after amphetamine challenge. This period is not enough to get a significant FosB protein translation. Taking into account the fact that FosB (42 kDa) is heavier than its isoform ΔFosB (35~37 kDa) (Kovács, 1998; Nestler et al., 2001). We measured only proteins with 37 kDa of molecular weight.

Equal protein loading was confirmed by stripping the blots and re-probing them with a monoclonal beta-actin antibody (loading control) (1:500; Sigma-Aldrich), followed by incubation with respective secondary antibody (Cy5—anti-rabbit/1:3000) and visualization as described above. The intensity of ΔFosB protein band was divided by the intensity of the internal loading control (beta-actin) for that sample. The ratio of ΔFosB to loading control was then used to compare ΔFosB abundance in different samples (Figure 2B).

### Statistical Analysis

All data are expressed as mean ± SEM. Levene tests for homogeneity of variance were performed to the behavioral and molecular data. Levene did not show statistically significant differences for behavioral or molecular data, indicating the homogeneity of variance. Thus locomotor activity, ΔFosB levels following saline or amphetamine injections were analyzed using a 2 × 2 ANOVA [stress (repeated restraint or non-stress) × drug treatment (AMPH or SAL)]. When a significant (*p* < 0.05) main effect was observed, the Newman-Keuls test was used for *post hoc* comparisons.

## Results

### Stress-Induced Cross-Sensitization to Amphetamine

In this experiment, we assessed whether exposure to repeated stress could increase the locomotor response to an amphetamine challenge injection.

**Figure 1:**
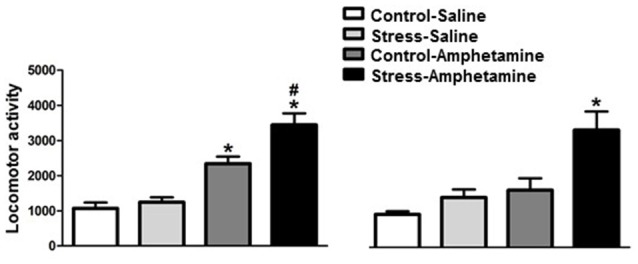
**Cross-sensitization between stress and amphetamine in adult and adolescent rats.** *p < 0.05 compared to CONTROL-SALINE and STRESS-SALINE; ^#^*p* < 0.05 compared to CONTROL-AMPHETAMINE.

We found that in adult rats, differences in amphetamine-induced locomotion are considered both stress (*F*_(1,29)_ = 7.77; *p* < 0.01) and treatment (*F*_(1,29)_ = 57.28; *p* < 0.001) factors. The interaction between factors was also detected (*F*_(1,29)_ = 4.08; *p* < 0.05; Figure 1). Further analysis (Newman-Keuls test) revealed that amphetamine administration increased locomotor activity in both control and stressed animals when compared to control and stress saline-injected animals. Furthermore, rats that were repeatedly exposed to restraint stress showed significantly higher amphetamine-induced locomotor activity when compared to the amphetamine control group (*p* < 0.05, Figure 1).

In adolescent rats, we found differences within both stress (*F*_(1,25)_ = 11.58; *p* < 0.01) and treatment (*F*_(1,25)_ = 16.34; *p* < 0.001) factors. However, no interaction between factors was detected (*F*_(1,25)_ = 3.67; *p* = 0.067; Figure 1). Further analysis (Newman-Keuls test) on the treatment factor revealed that amphetamine increases locomotor activity in stressed, but not control, animals when compared to saline-injected animals. Additionally, rats repeatedly exposed to restraint stress showed significantly higher amphetamine-induced locomotor activity when compared to the amphetamine control group (*p* < 0.01, Figure 1).

### Western Blotting Analysis ΔFosB Expression

We performed this experiment to evaluate whether the behavioral cross-sensitization induced by repeated restraint stress and amphetamine challenge could be related to alterations in ΔFosB expression on nucleus accumbens of rats in different development periods.

**Figure 2:**
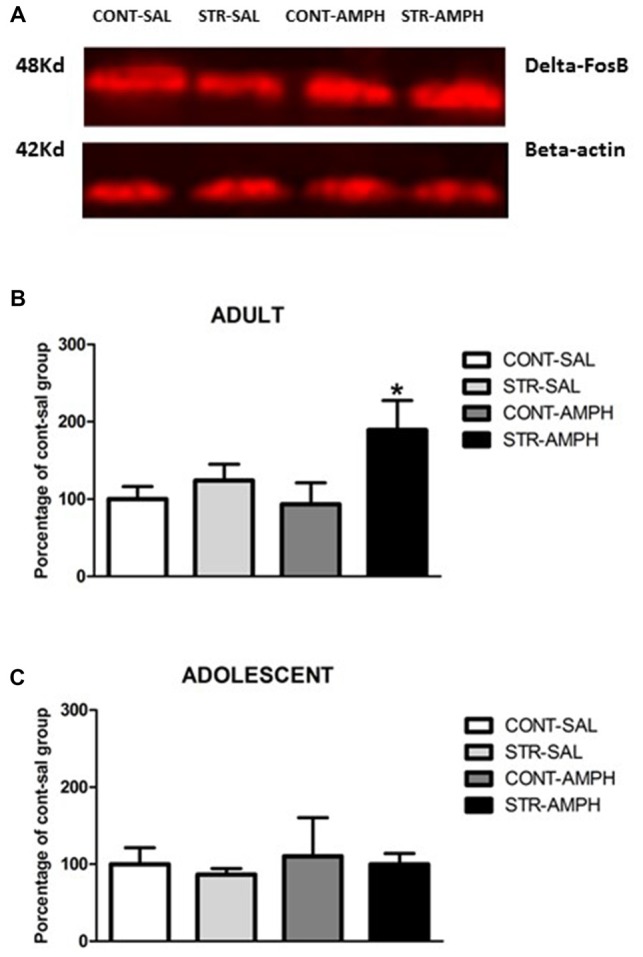
**(A)** Schematic section of the rat brain, adapted from the stereotaxic atlas of Paxinos and Watson (2006), showing the location of the punches in the nucleus accumbens (Nac). **(B)** Representative western blotting bands of control-saline (CONT-SAL), stress-saline (STRESS-SAL), control-amphetamine (CONT-AMPH) and stress-amphetamine (STRESS-AMPH) adult animals. **(C)** ΔFosB levels in response to amphetamine (1.0 mg/kg) or saline after repeated stress exposure in the nucleus accumbens of adult and adolescent rats. The bars represent mean ± SEM of 6–9 animals per group. *p < 0.05 different than other groups.

In adult rats, we observed significant differences in stress factor (*F*_(1,18)_ = 6.46; *p* < 0.05) and the interaction between stress and treatment factors (*F*_(1,18)_ = 5.26; *p* < 0.05). Further analysis (Newman-Keuls test) revealed that amphetamine increased ΔFosB levels in stressed animals compared to all other groups (*p* < 0.05, Figure 2C).

For adolescent rats, our results did not show any differences among the groups (Figure 2C).

## Discussion

We assessed the level of ΔFosB, on accumbens of adult and adolescent rats following the chronic stress induced locomotor cross-sensitization with amphetamine. The experiments highlights were: (a) adult and adolescent rats exhibited increase in locomotor activity after amphetamine challenge, induced by exposure to repeated stress; (b) repeated stress promoted increase in ΔFosB levels only on nucleus accumbens of adult rats.

Our data showed that stress-induced cross-sensitization to amphetamine in both adult and adolescent rats. These findings are in agreement with other studies, which show that repeated stress experiences result in cross-sensitization to psychostimulants in both adult (Díaz-Otañez et al., 1997; Kelz et al., 1999; Colby et al., 2003; Miczek et al., 2008; Yap and Miczek, 2008) and adolescent rodents (Laviola et al., 2002). Indeed, we have already demonstrated that adolescent and adult rats repeatedly exposed to chronic restraint showed a significant increase in locomotor activity after a challenge dose of amphetamine 3 days after the last stress session as compared to their respective saline controls (Cruz et al., 2012). Although many studies have shown cross-sensitization in stressed adult and adolescent rats challenged with psychostimulants, the underlying mechanisms are not well known yet.

We observed that stress-induced sensitization to amphetamine was associated with increased expression of ΔFosB levels in the nucleus accumbens in adults, but not in adolescent rats. Our finding expands previous data from the literature showing enhancement in expression of ΔFosB in response to psychostimulants after exposure to repeated stress in adult rats (Perrotti et al., 2004). Our results may suggest that increased ΔFosB levels could enhance the sensitivity to amphetamine in adult rats. Indeed, it was demonstrated that overexpression of ΔFosB within the nucleus accumbens increases the sensitivity to the rewarding effects of cocaine (Perrotti et al., 2004; Vialou et al., 2010). However, our finding implies association only. Functional studies have to be conducted to assess the causal of ΔFosB in stress-induced locomotor cross-sensitization to amphetamine.

Evidence suggests that ΔFosB is an important transcription factor which can influence the addiction process and could mediate sensitized responses to drug or stress exposure (Nestler, 2008). Studies have shown prolonged induction of ΔFosB within the nucleus accumbens in response to chronic administration of psychostimulant or different forms of stress (Hope et al., 1994; Nestler et al., 1999; Perrotti et al., 2004; Nestler, 2015). The importance of ΔFosB in the development of compulsive use of drugs may be due to its ability to increase the expression of proteins that are involved in the activation of the reward and motivation system (for review see McClung et al., 2004). For example, ΔFosB seems to increase the expression of glutamatergic receptors in the accumbens, which has been correlated with increasing the rewarding effects of psychostimulants (Vialou et al., 2010; Ohnishi et al., 2011).

Our adolescent data corroborate some studies which demonstrated that restraint stress or amphetamine administration induced behavioral sensitization to amphetamine without affecting ΔFosB expression in nucleus accumbens (Conversi et al., 2008). In the same way, Conversi et al. (2011) observed that although amphetamine has induced locomotor sensitization in C57BL/6J and DBA/2J mice, ΔFosB was increased in the nucleus accumbens of C57BL/6J, but not of DBA/2J sensitized mice. Taken together, these studies suggest that accumulation of ΔFosB in nucleus accumbens is not essential for the expression of locomotor sensitization. Thus, increase in the expression of this protein, as found in some studies, may be just a correlational observation.

Lower dopamine levels in the synaptic and a reduced dopaminergic tone, which is observed in adolescent rodents may perhaps justify the alterations in ΔFosB in the nucleus accumbens after prolonged stress exposure in adolescent rats, since the activation of dopaminergic receptors has been shown to be essential in increase in accumulation of ΔFosB in the nucleus accumbens after repeated psychostimulant administration (Laviola et al., 1999; Tirelli et al., 2003).

In conclusion, repeated restraint stress increased amphetamine-induced locomotion in both the adult and adolescent rats. In addition, stress and amphetamine seems to alter transcription of ΔFosB in an age-dependent manner.

## Author Contributions

Experiments were planned by PECO, PCB, RML, FCC and CSP, carried out by PECO, PCB, RML, FCC, MTM and the manuscript was written by FCC, PECO, PCB, RML and CSP.

## Conflict of Interest Statement

The authors declare that the research was conducted in the absence of any commercial or financial relationships that could be construed as a potential conflict of interest.
